# Generation and analysis of expressed sequence tags from NaCl-treated *Glycine soja*

**DOI:** 10.1186/1471-2229-6-4

**Published:** 2006-02-22

**Authors:** Wei Ji, Yong Li, Jie Li, Cui-hong Dai, Xi Wang, Xi Bai, Hua Cai, Liang Yang, Yan-ming Zhu

**Affiliations:** 1 Plant Bioengineering Laboratory, Northeast Agricultural University, 150030 Harbin, China

## Abstract

**Background:**

Salinization causes negative effects on plant productivity and poses an increasingly serious threat to the sustainability of agriculture. Wild soybean (*Glycine soja*) can survive in highly saline conditions, therefore provides an ideal candidate plant system for salt tolerance gene mining.

**Results:**

As a first step towards the characterization of genes that contribute to combating salinity stress, we constructed a full-length cDNA library of *Glycine soja *(50109) leaf treated with 150 mM NaCl, using the SMART technology. Random expressed sequence tag (EST) sequencing of 2,219 clones produced 2,003 cleaned ESTs for gene expression analysis. The average read length of cleaned ESTs was 454 bp, with an average GC content of 40%. These ESTs were assembled using the PHRAP program to generate 375 contigs and 696 singlets. The resulting unigenes were categorized according to the Gene Ontology (GO) hierarchy. The potential roles of gene products associated with stress related ESTs were discussed. We compared the EST sequences of *Glycine soja *to that of Glycine max by using the blastn algorithm. Most expressed sequences from wild soybean exhibited similarity with soybean. All our EST data are available on the Internet (GenBank_Accn: DT082443~DT084445).

**Conclusion:**

The *Glycine soja *ESTs will be used to mine salt tolerance gene, whose full-length cDNAs will be obtained easily from the full-length cDNA library. Comparison of *Glycine soja *ESTs with those of Glycine max revealed the potential to investigate the wild soybean's expression profile using the soybean's gene chip. This will provide opportunities to understand the genetic mechanisms underlying stress response of plants.

## Background

Environmental factors that impose water-deficit stress, such as drought, salinity and extreme temperatures, place major limits on plant productivity [1]. It is a problem that deserves global attention. In particular, increasing soil salinization has necessitated the identification of crop traits/genes that confer resistance to salinity. Traditional breeding strategies are limited by the complexity of stress tolerance traits, low genetic variance of yield components under stress conditions and the lack of efficient selection techniques [2]. With the great progress of molecular biology, introducing some functional genes of interest to crop plants by genetic engineering seems to be a shortcut to improve stress tolerance [3]. However, the approach has been limited by the lack of understanding of metabolic flux, compartmentation and function [4]. Thus, the integrative, whole genome studies of various stress-resistant mechanisms are needed [5,6]. A series of functional genomics strategies have emerged as required and the applications of these new technologies will accelerate the relevant research.

Expressed sequence tags (ESTs), which are generated by large-scale single-pass sequencing of randomly picked cDNA clones, have proven to be an efficient and rapid means to identify novel genes [7]. With many large-scale EST sequencing projects in progress and new projects being initiated, comparative genomics approaches are needed to assign putative functions to these cDNAs [8]. Such studies will present opportunities to accelerate progress towards understanding the genetic mechanisms underlying stress response of plants.

*Glycine soja *(50109) is one of the highly salt tolerant species that grows in coastal regions. The seeds were found to tolerate up to 0.9% of salt during germination stage, while *Glycine max *cannot grow well in regions where the salt concentration is 0.3% [9]. It is thus an ideal candidate plant for mining salt-tolerance genes.

In this study, single-pass sequences of randomly selected cDNA clones from a full-length cDNA library of *Glycine soja *leaf treated with 150 mM NaCl were obtained. The ESTs were classified into functional categories through comparisons with *Glycine max*, *Arabidopsis *and *Oryza sativa *genes in known databases. The potential roles of gene products associated with stress related ESTs were discussed.

## Results and discussion

### Generation of ESTs from *Glycine soja *subjected to salt stress

**Figure 1:**
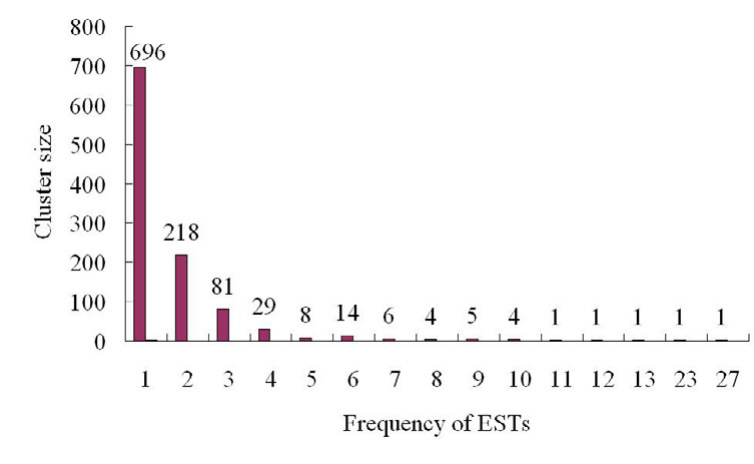
Distribution and number of clustered sequences.

The information provided by ESTs of randomly isolated gene transcripts generated under specific abiotic stress conditions provides an opportunity for gene discovery in addition to identifying the biochemical pathways involved in plant physiological responses [10]. Here, we describe ESTs obtained from salinity-induced cDNA library prepared from the leaves of the *Glycine soja *exposed to stress for a short period of time. Insert amplification of all random clones from cDNA library revealed inserts ranging between 500 bp and 2000 bp, with an average size of 1250 bp. A total of 2,219 clones were sequenced, and 2,003 cleaned EST sequences were generated for further analysis after trimming off vector sequences and removing of sequences shorter than 100 bp (GenBank_Accn: DT082443~DT084445). The average read-length of cleaned ESTs was 454 bp. The cleaned ESTs include 1936 5'end sequences and 67 3'end sequences (Table 1). The average G+C content of *Glycine soja *ESTs was 40%, which is similar to that of soybean [11]. The 2003 ESTs were assembled into 375 contigs and 696 singlets (clusters) using the PHRAP program (Table 1). The frequency of EST distribution after clustering is shown in Fig. 1. Nine contigs had 10 or more ESTs, with the largest one containing 27 ESTs. Most contigs contained one to six ESTs. The redundancy level of EST collection was 65%, which means that continued sequencing of cDNAs selected at random from our libraries still has considerable potential to uncover novel sequences.

### Comparisons of *Glycine soja *ESTs with those in Glycine max, Arabidopsis and Oryza sativa

Blastn was used to compare the EST sequences of *Glycine soja *to *Glycine max*, Arabidopsis and rice. The E-value was set at 1e-30. Although the size of *Glycine max *Gene Index is smaller than the AGI and OGI, the sum of matching section between *Glycine soja *and *Glycine max *(3106) was far more than *Glycine soja *versus *Arabidopsis *or *Glycine soja *versus *Oryza sativa *(Table 2). Note that there is great difference in stress-tolerant characteristics between soybean and wild soybean, although they share a large amount of homologs in expressed sequences. This indicates that the discrepancy in stress responses may come from the subtle difference between the homologous sequences. It is therefore feasible to investigate the wild soybean's gene expression profile using the Affymetrix soybean chip.

In order to get more information about the expression pattern of *Glycine soja *ESTs, BLASTN was used to search against the *Arabidopsis *CDS from TAIR, and 244 ESTs were highly similar to genes from *Arabidopsis*. The corresponding *Arabidopsis *genes were searched for the expression data under salt stress since global expression profiling of the *Arabidopsis *was available from TAIR[12]. As a result, a total of 126 ESTs were predicted to be up-regulated in response to salt stress according to AtGenExpress, and may be induced by salt stress. This prediction will be confirmed by further analysis.

### Functional categorization of *Glycine soja *ESTs and Putative stress-regulated genes

**Figure 2:**
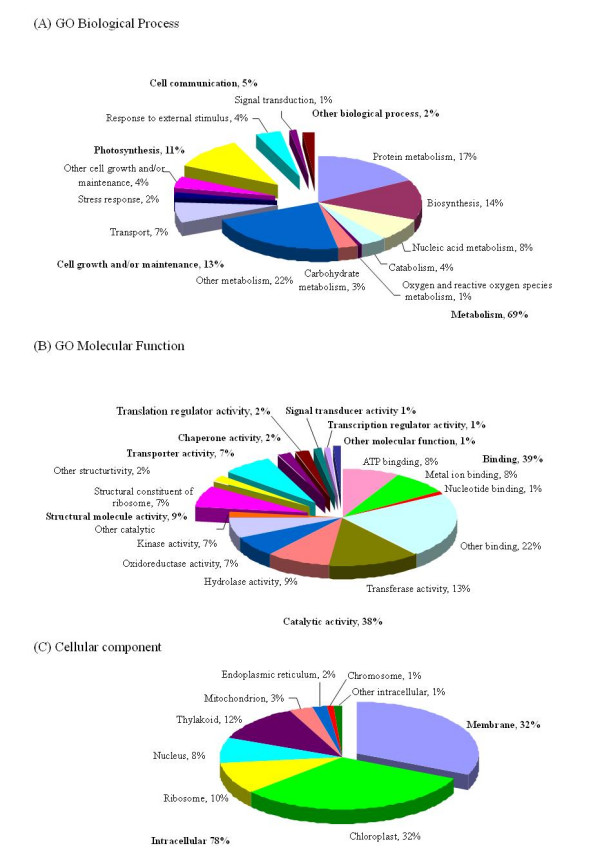
Representation of Gene Ontology (GO) mapping results for *Glycine soja *non-redundant ESTs.

As shown in Tables 3 and Figure 2, all unigenes were classified according to terms of biological processes, molecular functions and cellular components, developed by the Gene Ontology Consortium [13] in Uniprot (EBI). These genes cover a broad range of the GO functional categories. However, due to the lack of gene products information, many transcripts cannnot be functionally categorized. These 'unknown' genes are likely the source of candidate salt-tolerant genes and further functional analysis will help elucidate their specific roles in salt tolerance [14].

We successfully classified 279 unigenes in terms of biological processes (Fig. 2A), 301 unigenes in terms of molecular function (Fig. 2B), and 262 unigenes in terms of cellular components. Since one gene product may be assigned to more than one GO terms, and one children term can fit into multiple parental categories, the total number of GO mappings in each of the three ontologies will exceed the number of genes.

A large proportion of genes were found to participate in the biological process of metabolism (69%), followed by cell growth and/or maintenance (13%). The accumulation of osmoprotectants by either altering metabolism or increasing transport is an important process of plants for the adaptation to environmental stress [15]. It has been reported that in Arabidopsis, salinity induces programmed cell death in primary roots and the plants produce secondary roots which function better under abiotic stress [16]. The increase in metabolism could be essential to nutrient redistribution and new tissue development, a strategy the plants adopted to cope with the changed environment.

Our results showed that 4% of the unigene set responds to external stimulus, while 2% responds to stress (Fig. 2A). These two catgories form the basis for mining the stress-regulated genes. Genes encoding dehydration-induced ERD15 protein (DT083772), late embryogenesis abundant (LEA) protein (DT084384) and other stress-induced proteins were found in these categories. Submergence induced gene, induced by anaerobic stress, was also found in the ESTs sequenced (DT082680). There were also other genes function as scavengers of reactive oxygen species, such as catalase, glutathione S-transferase, and superoxide dismutase. These gene products are needed to maintain the redox homeostasis under abiotic stress. It was reported that overexpression of H_2_O_2_-scavenging enzymes increased the tolerance of plants to abiotic stress[17]. Metallothioneins (MT) are a group of low-molecular-weight (LMW) metal-binding proteins with a high cysteine content that are thought to be involved in metal ion metabolism and detoxification [18]. MT-like transcripts have been reported to be highly up-regulated in response to salt stress in barley [19,20]. Type 2 metallothionein (DT083320, DT083023) was present in our database.

In addition, proteins involved in the regulation of signal transduction pathway (Fig 2B) have been categorized separately. In plant cells, calcium functions as a second messenger coupling a wide range of extracellular stimuli to intracellular responses [21]. Calmodulin, one major class of Ca^2+ ^sensor characterized in plants, which was present in the *Glycine soja *ESTs (DT083725), is involved in stress signal transduction suggested by several lines of evidence [21-23].

Genes for transcription factors that contain typical DNA binding motifs, such as MYB, bZIP, have been demonstrated to be stress inducible [24]. Transcription factors containing similar domains are present in the *Glycine soja *ESTs and may be important in regulating the response to salt stress.

## Conclusion

We sequenced 2003 ESTs generated from salinity-treated *Glycine soja *cDNA library, putatively representing 1071 unigenes. Comparison of *Glycine soja *ESTs with those of Gl*ycine max *revealed the potential to investigate the wild soybean's expression profile using the soybean's gene chip. Through analysis of the ESTs with putative functional annotations, a large number of putative stress-regulated genes were identified. The full-length cDNAs of these genes can be obtained easily and their specific functions in salt tolerance can be further investigated using transformation technology in model systems, which will eventually provide new gene targets for the genetic engineering of other crop plants for improved resistance to abiotic stresses. Our results will also facilitate genomic analysis in other plant systems.

## Methods

### Plant materials

Seeds of *Glycine soja *(50109) were inoculated in half-strength solid MS medium (pH5.8) in the dark until germination. Plants were grown at 25°C in a greenhouse with a photoperiod of 15 h light/9 h dark. One-month-old seedlings were transferred into 150 mM NaCl solutions. Equal leaves were sampled at 0.5 h, 1 h, 3 h and 6 h and immediately frozen in liquid nitrogen. Frozen tissues were stored at -80°C until use.

### RNA preparation and construction of full-length cDNA library

Total RNA was isolated from plant materials with Trizol (Invitrogen) according to the manufacturer's instructions. The RNA concentration was determined by spectrophotometry, and its integrity was assessed by electrophoresis in 1% (w/v) formaldehyde-agarose gels [25].

For the full-length cDNA library, 2 μg of mRNA were used for cDNA synthesis using the SMART cDNA synthesis kit (Clontech, Palo Alto, CA, USA) according to the manufacturer's protocol. The resulting double-stranded cDNAs were digested with SfiI and ligated into the SfiI site of λ TriplEx2. The phagemids were packaged according to the instruction of Gigapack III Plus-7 packaging extract kit (Stratagene company). The average titer of the libraries was ~2 × 10^5 ^pfu/ml.

### Template preparation and DNA sequencing

Homologous recombination with E. coli BM25.8 was conducted to convert the phage libraries to the plasmid form. 8300 colonies were randomly selected and activated as templates of PCR reactions. The primers are as follows: P5':5'-GGCCATTACGGCCGGG-3'; P3':5'-CCGAGGCGGCCGACATG-3'. PCR was performed for 30 cycles of 30 s at 94°C, 30 s at 69°C and 2 min at 72°C. The PCR products were electrophoresed next to DNA size markers to estimate the molecular sizes of the insert DNAs. The clones with inserted fragments' size ≥ 500 bp were sequenced by Shanghai Sangon Company.

### Sequence analysis

The trimming process, which included the removal of low-quality sequences, poly(A) tails, ribosomal RNA, and vector regions, was conducted as described by Telles and da Silva [26] with minor modifications. In addition, sequences shorter than 100 bases were not included in the analysis.

The resulting sets of cleaned sequences were assembled into contigs by PHRAP program[27] using the following parameters: minmatch 100, minscore 94.

To assign annotation to contigs, BLASTX was used to search the Uniprot (EBI) with terms from the Gene Ontology Consortium[28] controlled vocabularies. The expectation value (e-value) cutoff for BLASTX was set at 1e-5.

In order to survey the similarity between soybean and wild soybean expressed sequences, our set of ESTs was blasted against local installations of GMGI (*Glycine max *Gene Index, release 12), AGI (*Arabidopsis *Gene Index, release 12) and OGI (*Oryza sativa *Gene Index, release 16) from TIGR. The *Glycine soja *ESTs were also blasted against *Arabidopsis *CDS from TAIR (release 6) at 1e-15. The raw data (cel file) of microarray experiment of *Arabidopsis *from TAIR (AtGenExpress) were used to identify up-regulated CDS of *Arabidopsis *response to salt stress. The software RMAExpress (Ben Bolstad) was used to scale/normalize the raw data.

## Authors' contributions

The first four authors contributed equally to this work. Wei Ji participated in the EST data analysis and drafted the manuscript. Yong Li performed the data analysis and helped to draft the manuscript, and is one of the co-first authors. Jie Li participated in the planning and supervising of the study, and is one of the co-first authors. Cui-hong Dai participated in construction of full-length cDNA library and template preparation, and is one of the co-first authors. Yan-ming Zhu participated in the design of the study, and is the corresponding author. All authors read and approved the final manuscript.

## Figures and Tables

**Table 1:** *Glycine soja *EST Summary

Total ESTs	2219
Total high-quality ESTs	2003
Success index (%)	90.3
5'-end sequences	1936
3'-end sequences	67
Average insert size (bp)	1250
Average sequence size (bp)	454
Average GC content(%)	40
Number of contigs	375
Number of singlets	696
Number of unigenes	1071

**Table 2:** Comparison of *Glycine soja *ESTs with those in *Glycine max*, *Arabidopsis *and *Oryza sativa*

Database	Number of bp	Number of sequences	E-value	Sum of matching section	Matching summary	Average matching length
AGI	62,362,651	61,603	1e-30	235	≥ 98% 9 ≥ 90% 89	≥ 200 bp
GMGI	37,918,896	63,676	1e-30	3106	≥ 98% 1011 ≥ 90% 2078	≥ 200 bp
OGI	93,862,193	89,147	1e-30	521	≥ 98% 57 ≥ 90% 391	≥ 200 bp

**Table 3:** The GO categorization of *Glycine soja *ESTs by biological process, molecular function, and cellular component

	Gene Ontology term	Representation	Representation percentage
Biological process	Metabolism	385	69%
	Protein metabolism	92	17%
	Biosynthesis	79	14%
	Nucleic acid metabolism	44	8%
	Catabolism	24	4%
	Oxygen and reactive oxygen species metabolism	4	1%
	Cell growth and/or maintenance	70	13%
	Transport	37	7%
	Stress response	10	2%
	Photosynthesis	63	11%
	Cell communication	27	5%
	Response to external stimulus	20	4%
	Signal transduction	5	1%
	Developmental process	2	<1%
	Cell death	2	<1%
Molecular function	Binding	202	39%
	ATP bingding	43	8%
	Metal ion binding	41	8%
	Nucleotide binding	7	1%
	Catalytic activity	197	38%
	Transferase activity	67	13%
	Hydrolase activity	45	9%
	Oxidoreductase activity	38	7%
	Kinase activity	34	7%
	Structural molecule activity	45	9%
	Structural constituent of ribosome	38	7%
	Transporter activity	37	7%
	Chaperone activity	10	2%
	Translation regulator activity	8	2%
	Signal transducer activity	4	1%
	Transcription regulator activity	4	1%
	Enzyme regulator activity	1	<1%
	Motor activity	1	<1%
Cellular component	Intracellular	332	73.3%
	Membrane	121	26.7%
